# Longitudinal borehole functionality in 15 rural Ghanaian towns from three groundwater quality clusters

**DOI:** 10.1186/s13104-022-05998-1

**Published:** 2022-03-22

**Authors:** Olivia L. Schultes, Mustafa Sikder, Emmanuel A. Agyapong, Michelle O. Sodipo, Elena N. Naumova, Karen C. Kosinski, Alexandra V. Kulinkina

**Affiliations:** 1 School of Arts and Sciences, Tufts University, Medford, MA USA; 2 University of Washington School of Public Health, Seattle, WA USA; 3 School of Engineering, Tufts University, Medford, MA USA; 4 Institute for Health Metrics and Evaluation, Seattle, WA USA; 5 University College of Agriculture and Environmental Studies, Bunso, Ghana; 6 Harvard T.H. Chan School of Public Health, Boston, MA USA; 7 Friedman School of Nutrition Science and Policy, Tufts University, Boston, MA USA; 8 Swiss Tropical and Public Health Institute, Basel, Switzerland; 9 University of Basel, Basel, Switzerland

**Keywords:** Boreholes, Functionality, Water quality, Distance, Payment, Ghana

## Abstract

**Objective:**

In sub-Saharan Africa, 45% of the rural population uses boreholes (BHs). Despite recent gains in improved water access and coverage, parallel use of unimproved sources persists. Periodic infrastructure disrepair contributes to non-exclusive use of BHs. Our study describes functionality of BHs in 2014, 2015, and 2016 in 15 rural towns in the Eastern Region of Ghana sourced from three groundwater quality clusters (high iron, high salinity, and control). We also assess factors affecting cross-sectional and longitudinal functionality using logistic regression.

**Results:**

BH functionality rates ranged between 81 and 87% and were similar across groundwater quality clusters. Of 51 BHs assessed in all three years, 34 (67%) were consistently functional and only 3 (6%) were consistently broken. There was a shift toward proactive payment for water over the course of the study in the control and high-salinity clusters. Payment mechanism, population served, presence of nearby alternative water sources, and groundwater quality cluster were not significant predictors of cross-sectional or longitudinal BH functionality. However, even in the high iron cluster, where water quality is poor and no structured payment mechanism for water exists, BHs are maintained, showing that they are important community resources.

## Introduction

In sub-Saharan Africa, 59% of the population lives in rural areas [1] and 45% is estimated to use boreholes (BHs) [2]. In Ghana, according to the most recent Demographic and Health Survey, 45% of rural residents reported primarily using BH water for drinking, while 20% primarily used unimproved sources, such as unprotected hand-dug wells, unprotected springs, or surface water [3]. Despite recent gains in improved water access and coverage, parallel use of improved and unimproved sources persists, especially in rural areas [3–5]. Periodic infrastructure disrepair contributes to non-exclusive use of BHs; at any given time, approximately 26% of BHs in Ghana are broken [6]. The connection between poor health outcomes and insufficient water, sanitation, and hygiene (WASH) infrastructure has been recently reviewed [7].

Predictors of BH functionality have been evaluated using primarily cross-sectional studies. Improved functionality has been associated with access to pump spare parts and skilled labor [8, 9], presence of a payment scheme [8, 9], and external post-construction support programs [5]. Reduced functionality was associated with BH installation during the rainy season [10], infrastructure age [8], and longer distances to a major city [9]. The presence and number of alternative water sources in a community has also been negatively associated with BH functionality [5, 8].

Although inherently a dynamic concept, a relatively small number of studies have attempted to capture BH functionality over time, primarily through retrospective key informant interviews [8, 9, 11–13]. Qualitative information on historical functionality was used in conjunction with a single observed functionality measure. A key gap in the literature is the lack of longitudinal BH functionality data in low-income countries; this is partly due to fragmented systems for BH construction and monitoring and the resource-intensive nature of repeated field observations [14, 15].

We conducted several prior studies in the Eastern Region of Ghana investigating water infrastructure. The first study showed that groundwater access in the area is abundant, with 87–95% of the population having access to a borehole within 500 m. Despite this, surface water continues to be used extensively [16]. The second study identified three distinct spatial groundwater quality clusters: high iron, high salinity, and a control area with no detected water quality problems [17]. Our third study showed that households in the high iron and high salinity clusters were significantly more likely to use surface water as compared to the control cluster [4].

Following on the aforementioned prior studies, the present study describes longitudinal functionality of BHs over the course of three years in 15 rural Ghanaian towns. We use logistic regression to explore common factors affecting functionality, such as payment mechanism, population served, presence of alternative water sources, and groundwater quality. The primary contributions of the study are the longitudinal quantitative functionality data and contextual knowledge from three years of monitoring.

## Main text

### Methods

#### Study setting

This study was conducted in the Eastern Region of Ghana. We investigated BH functionality over three years in the same 15 towns as the water use study [4], distributed among three groundwater quality clusters: high iron, high salinity, and control. The high iron and high salinity clusters corresponded to the Upper Birimian and granites and granodiorites geological formations, respectively [17]. The towns ranged in population between 829 and 3347 people and relied on a combination of BHs, hand-dug wells and surface water. Further details on the study towns can be found in a prior publication [4].

#### Data collection

We collected several types of data about public water sources in January 2014, May 2015, and May 2016 (Table 1). The geographic locations of water features (BHs, hand-dug wells, surface water access points) were recorded using a handheld GPS device (Garmin GPS 165 72H Portable Navigator, Garmin, Ltd.) or the iPad GPS Tracks app (version 2.8.7). BH functionality was determined by manually testing each BH to see if it produced water. Functionality status was also ascertained with nearby residents. BH water quality was tested in 2015 by a certified water quality laboratory [17]. BH payment method was collected using an open-ended question, triangulated between nearby residents and the water committee members, and subsequently categorized (Table 1).

**Table 1:** Summary of data collected in 2014, 2015, and 2016 and analysis variables

Data collected	2014	2015	2016
Borehole GPS coordinates	✓	✓	✓
Hand-dug well GPS coordinates	✓		✓
Surface water access point GPS coordinates	✓		✓
Household GPS coordinates			✓
Borehole functionality	✓	✓	✓
Borehole water quality		✓	
Borehole payment method	✓		✓

Analysis variable	Type	Definition	
Functionality status	Binary	1 = functional; 0 = not functional	
Groundwater quality cluster	Categorical	control = no identified water quality problems; high salinity = elevated total dissolved solids concentration; high iron = elevated iron concentration	
Payment mechanism	Binary	1 = proactive; 0 = reactive or none	
Population served within 300 m	Binary	1 = 600 + people; 0 = ≤ 600 people	
Another water source within 300 m	Binary	1 = present; 0 = absent	

#### Data analysis

We derived several analysis variables from the available data (Table 1). Groundwater quality clusters were allocated based on spatial interpolation of water quality results, namely iron and total dissolved solids concentrations [17]. Reported payment methods fell into one of three categories: proactive (per volume or unit of time), reactive (per household once the BH is already broken), or no payment. Reactive and no payment were combined to form a binary payment variable (Table 1). A service area was delineated as a 300-m Euclidean buffer distance around each BH. Population served was calculated as the number of households within the buffer area (mapped in 2016), multiplied by the average household size of 3.5 [3], and categorized (Table 1). Lastly, we created a binary variable for whether there was another water source (e.g., another BH, hand-dug well or surface water source) available within the 300 m buffer distance of each BH. All geospatial analyses were conducted in ArcGIS software (version 10.5.1).

We applied univariable and multivariable logistic regression models to assess the association between BH functionality and the four explanatory variables in each of the three study years. A fourth model used the same explanatory variables on a dataset of BHs that were examined in all three time points (2014, 2015, and 2016). For the fourth model, longitudinal functionality status was defined as 1 for BHs that were functional at all three time points and 0 for BHs that were non-functional at least once.

## Results

**Fig. 1:**
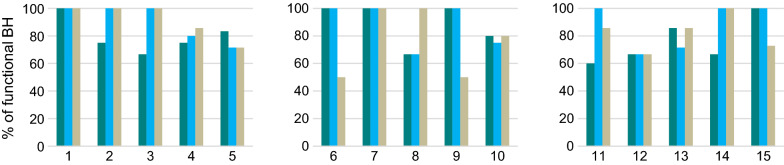
Borehole functionality (%) across study towns in control [towns 1–5 on the left], high salinity [towns 6–10 in the center], and high iron [towns 11–15 on the right] water quality clusters. Three bars for each town represent years (2014, 2015, and 2016) from left to right

Over the course of the study, we identified a total of 63, 67, and 73 BHs in the 15 towns in 2014, 2015, and 2016, respectively. Of these, 81%, 87% and 81% were functional in each year. Functionality rates were similar across the water quality clusters, but some towns (e.g., towns 4, 5, 10, 12) had notably lower functionality rates than others (Fig. 1). In 2014, most towns had a reactive or no payment mechanism for water (3/5 in the control and high salinity clusters and 5/5 in the high iron cluster). Between 2014 and 2016, four towns switched to a proactive payment mechanism (1 in control, 2 in high salinity and 1 in high iron). In 2016, most towns had a proactive payment mechanism, with reactive payment predominating only in the high iron cluster (4/5 towns). The average number of people per BH ranged between 272 and 1,200 (sd = 447) across study towns, with slightly higher numbers in the control cluster (p > 0.05).

A total of 51 BHs were present in all three datasets and used for assessing longitudinal functionality. Of these, 34 (67%) were functional during all three time points, 11 (21%) were broken during one time point, 3 (6%) were broken during two time points, and 3 (6%) were broken during all three time points. We tested each of the four explanatory variables to assess associations with cross-sectional and longitudinal BH functionality using univariable logistic regression (Table 2). None of the variables were significant predictors of BH functionality in any of the models. Multivariable logistic regression produced similar results (not shown), none of which were statistically significant. Payment mechanism and presence of another water source within 300 m were not evaluated in 2015 because they were not assessed in the field (Table 1).

**Table 2:** Odds ratios and 95% confidence intervals from univariable logistic regression models

	2014	2015	2016	Longitudinal
	n = 63	n = 67	n = 73	n = 51
Water quality cluster: high salinity	1.7 (0.3, 11.1)	0.9 (0.1, 6.5)	0.4 (0.1, 2.2)	1.1 (0.2, 6.0)
Water quality cluster: high iron	1.0 (0.3, 4.2)	1.1 (0.2, 5.3)	0.6 (0.1, 2.6)	0.8 (0.2, 3.1)
Payment mechanism: proactive	1.0 (0.2, 4.4)	–	3.4 (0.4, 30.1)	0.8 (0.2, 2.6)
Population served: > 600	0.2 (0.0, 1.7)	0.7 (0.1, 3.7)	1.1 (0.3, 3.9)	0.6 (0.2, 2.1)
Another water source: present	0.7 (0.1, 6.3)	–	0.8 (0.1, 7.7)	0.8 (0.2, 2. 6)

## Discussion

In this small-scale study, we found that 67% of the BHs observed at all three time points were consistently functional and only three BHs were consistently broken (one in each water quality cluster), showing a concerted effort to finance and repair BHs over time. We also found that some study towns switched from not paying for water at all or paying reactively when a BH breaks to a proactive payment mechanism over the course of the monitoring period. This shift was more common in the control and high salinity clusters. In the high iron cluster, four of five towns still were not charging for BH water in 2016. This is consistent with the contextual knowledge of our study team: residents of towns with elevated iron content are generally unwilling to pay for BH water and prefer to use free alternative water sources widely available in their neighborhoods. However, we anticipate that as household income and education levels increase over time, communities will transition to proactive payment mechanisms which facilitate sustained service, as these upstream factors are associated with access to improved drinking water in Ghana [18, 19].

While we hypothesized that groundwater quality, and therefore the level of satisfaction with BH water quality, would have a bearing on functionality, we found that in any given year, approximately 20% of the BHs were broken in the study towns, with no differences across clusters. Similar functionality rates were found in other studies conducted in rural Ghana [6, 8]. We also observed that the number of water users per BH was higher in the control cluster, as compared to towns with water quality problems. This observation is also consistent with the overall higher number of BHs in towns with water quality problems. In the case of the high iron cluster, iron concentrations in the water samples are more than ten times higher (up to 4.5 mg/L) than the water quality standard in Ghana (0.3 mg/L) [17]. We suspect that while BHs are being constructed, and water testing results reflect unsatisfactory quality, more BHs may be drilled to find a depth or location with lower iron levels. This may result in a higher number of BHs in these locations as compared to places where water quality standards are met.

We note that we intentionally excluded some important predictors that were significant in other studies: namely, the availability of and distance to BH mechanics and spare parts and presence of a water committee [13, 20]. Distance to BH mechanics and spare parts was excluded because all towns were located relatively close (< 25 km) to district capitals that had these resources, exhibiting no variability in the dataset. Presence of a water committee was excluded because it was highly correlated with proactive payment mechanism. Recent literature has considered local perception of water committees and the significant association with variables that capture aspects of community participation in Kenya [21], which represents an interesting angle for future study.

Historically, government resources in sub-Saharan African countries have been insufficient to repeat large-scale data collection efforts and track infrastructure over time [15], partly, due to legacies of colonialism still impacting present-day water systems [22, 23]. More recently, there is a shift toward mapping and monitoring of existing water supplies. For example, Liberia conducted extensive water point mapping in 2011 and 2017 (https://wash-liberia.org/raw-water-point-data/). The expansion of digital technologies, such as sensors on mechanized boreholes may provide further opportunity for cost-effective near real-time monitoring of water sources in remote and rural areas [24]. These systems may facilitate timely repairs when implemented in settings with sufficient financial and administrative support [24, 25], and may even be used to predict breakdowns [26]. Further availability of longitudinal data, new standardized data-sharing platforms, such as the Water Point Data Exchange (https://www.waterpointdata.org/), and advances in analytical methods [26–28] will allow governments, funding agencies, and implementing organizations to study infrastructure functionality and its correlates and direct resources toward sustainability.

## Conclusion

In our longitudinal dataset, payment method, population served, and presence of an alternative water source were not associated with BH functionality. Although organoleptic groundwater quality changed the water use patterns of the study communities [4], it was not correlated with BH functionality status over the 3-year monitoring period. Interestingly, in the high iron cluster, despite poor groundwater quality and lack of regular payment for water, the towns managed to maintain and repair their BHs, showing that they are important community resources, or potentially that they experience less heavy use. Our data set is from several years ago, but research on borehole sustainability in low-income settings remains limited. In combination with our prior studies, this dataset contributes to generating future hypotheses regarding the dynamic relationships among groundwater quality, functionality, use, and financial sustainability of rural water resources.

## Limitations

- The number of BHs sampled and the number of explanatory variables are relatively small, which may have led to null findings and limited generalizability.
- The length of the monitoring period was also short. More data points over a longer follow-up period would have enabled a more interesting longitudinal analysis using methods such as time series or survival analysis.

## Data Availability

The data are available from the corresponding author upon request.

## Abbreviations

BH: Borehole
GPS: Global positioning system
